# Parental Incarceration, Development, and Well-Being: A Developmental Systematic Review

**DOI:** 10.3390/ijerph20043143

**Published:** 2023-02-10

**Authors:** Alicia Herreros-Fraile, Rodrigo J. Carcedo, Antonio Viedma, Victoria Ramos-Barbero, Noelia Fernández-Rouco, Pilar Gomiz-Pascual, Consuelo del Val

**Affiliations:** 1 HIPRIFAM, Psychological Assistance for Children of Incarcerated Parents and their Families Unit, Faculty of Psychology, University of Salamanca, Avda. de la Merced 109-131, 37005 Salamanca, Spain; 2 Department of Developmental and Educational Psychology, Faculty of Psychology, University of Salamanca, Avda. de la Merced 109-131, 37005 Salamanca, Spain; 3 Department of Sociology I, Faculty of Political Sciences and Sociology, National Distance Education University (UNED), C/Obispo Trejo, 2, 28040 Madrid, Spain; 4 Health Sciences Department, Faculty of Health Sciences, University of Burgos, Paseo de los Comendadores, s/n (H. Militar), 09001 Burgos, Spain; 5 Department of Education, School of Education, University of Cantabria, Avda. de los Castros 50, 39005 Santander, Spain

**Keywords:** parental incarceration, development, well-being, children, adolescents, effects, moderators, mediators, systematic review

## Abstract

Despite an increasing number of studies examining the impact of parental incarceration on children’s well-being, there are few comprehensive reviews that collect this information, and even fewer from a developmental perspective. This study aims to clarify the effects of parental incarceration on children’s well-being and development, as well as the moderating and mediating factors from a developmental perspective. A systematic review was conducted according to PRISMA guidelines, selecting 61 studies of children from early childhood to adolescence. The results show differences in the current evidence regarding the effects of parental incarceration on children depending on the developmental stage, with the most evidence in the 7–11-year-old stage. Being male appears as a risk moderator factor while the mental health of the caregiver and their relationship with the child appears as a mediating variable, especially from 7 to 18 years old. These results reveal the impact of parental incarceration based on children’s age, providing a basis for developing protective and intervention measures.

## 1. Introduction

Imprisonment has a range of negative consequences on an individual’s personal life, and also on the families who are separated from the incarcerated people. This is especially relevant for the children of incarcerated parents. Having a parent in prison is a more common phenomenon than it may seem. Although there are no data or worldwide estimates, there are estimates that, in Europe, 723,000 children and adolescents are in this situation [1], and 2.6 million in the United States [2], the country with the highest incarceration rate in the world.

Over the past decade, several studies have been conducted, mainly in the United States [3], that have found significant associations between parental incarceration and different difficulties in the development of children of incarcerated parents, e.g., [4]. In this sense, children and adolescents have shown greater difficulties in their physical [5], cognitive [6] and socioemotional development [7,8,9], as well as in their school life [10], living conditions [11], and a greater number of psychosocial risks (e.g., substance abuse [12], delinquency [13], and violent behavior [9]). However, some studies have not found a relationship between parental incarceration and some of these variables, e.g., [14,15,16,17,18].

On the other hand, the target population differs depending on the research, with different studies focusing on the effects of parental imprisonment on children in early childhood [8], middle childhood [6], or adolescence [12]. It is essential to consider the child’s developmental stage as, depending on the child’s age, parental imprisonment may impact differently on the child’s well-being [19]. For example, parental incarceration may have a different impact on children’s academic performance depending on the stage of education. Parental incarceration has been more strongly associated with some developmental outcomes at one stage than others, e.g., [20]. Moreover, parental imprisonment may not only directly affect children’s well-being but may also affect children indirectly through mediating variables [10]. In addition, the consequences of imprisonment may also be modified through moderating variables [8]. Some characteristics of the family, the children themselves, the school, or the neighborhood where they live could be determinants of the relationship, modifying the effects of parental imprisonment or eliminating them [21]. For example, financial hardship and family circumstances are important mediators. In contrast, Child’s gender appears to be a significant moderator [22].

To date, several reviews and meta-analyses have explored the effects of parental imprisonment on physical health [21,23], mental health [23], and various other outcomes such as substance use, offending behavior, and educational performance [24,25,26]. However, these reviews do not present the results according to the developmental stage of the children, so it is not possible to observe whether the impact of imprisonment varies according to the age of the children. Nevertheless, the existence of different effects of parental imprisonment depending on the age of the children suggests the need to adopt a developmental perspective and consider life course patterns [27]. Implementing a developmental perspective when studying the impact of parental imprisonment on children is one of the lines of research that are still pending [19].

It has only been possible to locate two reviews that have analyzed the impact of parental imprisonment by separating children according to age [22,28]. On the one hand, Luk et al. [28] divide the included studies into children and adolescents. However, the group of “children” comprised ages ranging from birth to 11–12 years, a very long period in which the child undergoes many changes. Therefore, this review explores a further developmental stage by dividing the children group into early childhood and middle childhood. On the other hand, Poehlmann-Tynan and Turney [22] used these three developmental stages. Nevertheless, they do not set an inclusion criterion in the review to select only studies with a comparison group, as was the case with Luk’s review [28]. To observe the different effects of parental imprisonment on children, it is essential to compare these outcomes with those of other children who have not been exposed to parental imprisonment. It is the only way to determine whether parental imprisonment impacts children’s well-being and development. For this reason, this review not only explores in detail the effects of parental imprisonment in early childhood, middle childhood, and adolescence but carries out this analysis only through studies with a group of children with an imprisoned parent and a comparison group, unlike other reviews.

Furthermore, another problem identified with the analysis of this phenomenon lies in the methodological differences between studies on the effects of parental imprisonment. To make the results as comparable as possible, only quantitative articles have been selected. In addition, to show even more significant results, only those studies that include control variables have been considered. Control variables relate to characteristics before parental imprisonment likely to cause part of the effects to be studied. For example, children exposed to parental incarceration are three times more likely to suffer adverse childhood experiences than other children [29]. For this reason, it is essential to include only studies that controlled for a range of variables (socio-demographic, socio-economic, health, interpersonal, cognitive, and emotional) to ensure that the different outcomes observed in children were not solely dependent on other characteristics of children’s lives.

Finally, this review also addresses another of the lines of research pending to date, the in-depth analysis of the mediating and moderating variables of the relationship between parental imprisonment and different outcomes in children at different developmental stages [19]. These variables have yet to be rigorously analyzed in reviews with an evolutionary perspective, despite having been studied in some empirical research. In this sense, both Luk’s and Poelhmann-Tynan and Eddy’s reviews mention some mediators and moderators [22,28], but without examining them according to the age of the children not allowing us to observe the differences. This paper is the first review to analyze all the moderators and mediators analyzed in the included articles. The aim is to detect possible risk and protective factors as well as mechanisms that, depending on the age of the children, mediate or moderate the impact of parental imprisonment.

In summary, this fact leads us to propose the following research questions:

(1) Are there differences in the impact of parental imprisonment on children’s well-being and development depending on children’s developmental stage when a comparison group is included?
(2) What are the mediating and moderating variables in the relationship between parental imprisonment and the different outcomes related to children’s well-being and development at each developmental stage?

## 2. Materials and Methods: Search Strategy, Inclusion Criteria, and Research Article Selection

Databases related to this topic and of great relevance and prestige were used. Therefore, the databases PsycINFO, MEDLINE, ERIC, Web of Science, Scopus, and the Cochrane Library were used. The search was limited to the period 2000–2022 with the following search strategy: (incarcerated parents OR parental incarceration OR incarcerated mother* OR incarcerated father) AND ((impact on child* OR effect on child*) OR (child* socio-emotional development OR child* well-being OR child* wellbeing OR infant* socio-emotional development OR infant* well-being OR infant* wellbeing OR infant* health OR infant* quality of life)).

Regarding the criteria for selecting studies for inclusion in the review, the following were used:

- Participants’ age was between 0 and 18 years (except for studies that included older participants, provided the mean age of the sample was less than 18 years);
- The studies needed to assess the impact of parental incarceration on children and/or adolescents;
- One or both parents had to be or had been in prison;
- They needed to be quantitative studies and include groups of children with an incarcerated parent and a comparison group (without a parent in prison), in addition to control variables;
- The design could be cross-sectional or longitudinal;
- The studies had to be published in prestigious international journals (e.g., catalogued and/or indexed in PsycINFO, ERIC, Medline, Scopus, or Web of Science);
- The studies needed to be published in English or Spanish.

As an exclusion criterion, children could not live in the same prison as occurs in Mother Units. This profile is very different from children who live outside of prison with a caregiver.

**Figure 1:**
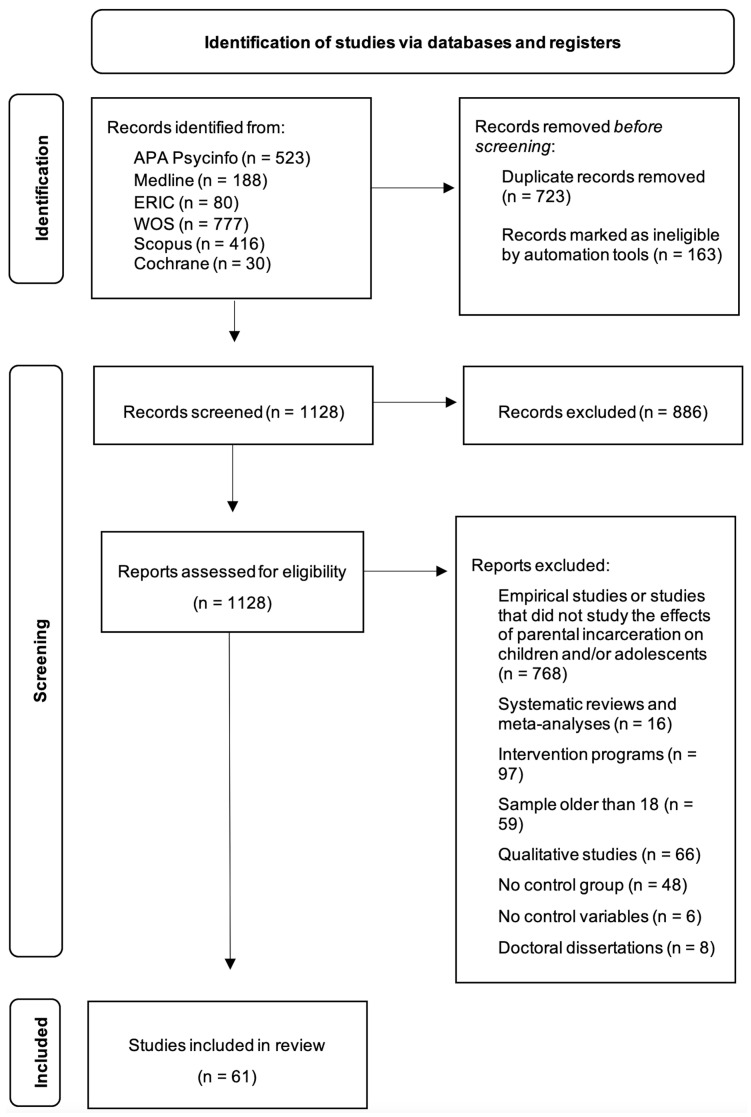
PRISMA flowchart detailing study selection process.

Article selection was performed following the procedure outlined in the PRISMA guidelines [30]. Below, we present the search flow diagram, Figure 1.

The initial search was performed with the previously mentioned search strategy in distinct databases: APA PsycInfo (n = 523), Medline (n = 188), ERIC (n = 80), Web of Science (n = 777), Scopus (n = 416), and Cochrane (n = 30) for a total of 2014 studies. Duplicate studies were eliminated manually and automatically by importing articles into the Mendeley platform, reducing the sample to 1128. Accordingly, from the resulting studies, those that did not meet the inclusion criteria on reading the abstracts or the full-length articles were eliminated. Firstly, studies that did not investigate the effects of parental incarceration in children and/or adolescents, or those that did not do so empirically were excluded (n = 768). Secondly, articles based on systematic reviews and meta-analyses (n = 16) and intervention programs (n = 97) were also not included. Thirdly, empirical studies with the following characteristics were also dropped from the final selection: samples older than 18 (n = 59), qualitative research (n = 66), quantitative research without a comparison group (n = 47), without control variables (n = 6), or theses or dissertations that were not published in a scientific journal (n = 8). After exclusion, 61 studies met all the inclusion criteria and were selected.

To perform data extraction and synthesis, the most important information was determined based on the research questions. In addition to the authors and the publication date, we extracted the age and size of the included sample, the variables or criterion variables of each study, the incarcerated parent (father, mother, or both), the type of study, the number of controlled variables (in intervals, with these being “between 1 and 5”, “between 5 and 10”, “between 10 and 15”, “between 15 and 20” and “more than 20”) and the results obtained. All this information was collected in four tables based on the age of the sample. Therefore, the sample was divided into four groups: children between 0 and 6 years old, children between 7 and 11 years old, adolescents between 12 and 18 years old, and a last group of those studies that did not differentiate developmental stages and that included boys and girls between 0 and 18 years. In the second stage, variables with a moderator or mediator effect in the relationship between parental incarceration and different outcomes related to the well-being and development of children were collected. These variables were included in this review if there was an appropriate statistical analysis of moderation or mediation. However, it is true that in the case of moderation indicated in the corresponding table, those studies that ran their analyses with separate groups but without using a moderation test were also considered.

## 3. Results

In this section, the direct effects of parental imprisonment on different outcomes, as well as the effects of moderators and mediators are presented in Table 1 and Table 2. Additionally, the results of each study included in this review may be consulted in Appendix A (Table A1, Table A2, Table A3 and Table A4).

### 3.1. Children 0 to 6 Years

Several studies have investigated the association of parental incarceration with physical health, cognitive skills and school performance, social–emotional skills, externalizing and internalizing symptoms, and material hardship in the early childhood stage.

#### 3.1.1. Physical Health

Four articles were included in this category, of which two differentiated between paternal and maternal incarceration [31,32]. Three of these studies found that parental incarceration was associated with greater sleep and eating behaviors problems [31], food insecurity [33], and infant mortality [32]. By contrast, one study did not find any significant relationship between parental incarceration and physical health, although this research used only one global health measure [8].

Regarding moderators, domestic violence by the father before incarceration moderated the relationship between the father’s incarceration and the child’s physical health (*p* < 0.10). Parents in prison who were not involved in domestic violence before imprisonment were associated with poorer physical health of their children [32]. On the other hand, although a formal analysis was not performed, paternal and maternal incarceration, analyzed separately, was associated with most physical health measures. While paternal incarceration was significantly associated with more sleep problems and starch consumption, maternal incarceration was associated with fast food consumption [31].

#### 3.1.2. Cognitive Skills and Academic Performance

A total of five studies were categorized within this section, three of which referred to paternal incarceration [8,10,34] and two which did not differentiate the gender of the parent in prison [16,35]. Parental incarceration was associated with lower cognitive skills and/or academic performance in four of these studies [8,10,34,35]. Those children with a parent in prison presented a greater number of attentional difficulties [8], were less prepared for school [34,35], and had a greater probability of repeating a grade [10]. In contrast, there was no association between parental incarceration and worse academic functioning [16]. Likewise, no significant results were found when studying the differences in children’s verbal ability [8].

Among the moderating and mediating variables considered, child’s gender appeared as a moderator, and the perception of teachers was a mediator. In this sense, paternal incarceration was significantly associated with a worse non-cognitive preparation for boys entering school but not girls [34]. Additionally, paternal incarceration was related to a more negative perception of teachers toward children, resulting in a deterioration in cognitive skills and school performance [10].

#### 3.1.3. Socioemotional Skills

Only one study examined how paternal incarceration affects socioemotional skills, specifically emotion recognition. The relationship was not significant [19].

#### 3.1.4. Externalizing Symptoms

Three studies analyzed the relationship between externalizing symptoms and parental incarceration, two of which referred to paternal incarceration [8,36] and one which did not differentiate the gender of the incarcerated parent [16]. Two of these studies found that children exposed to paternal incarceration were more likely to present more externalizing problems [8] and physical aggression behaviors [36]. On the other hand, another study found no significant results for externalizing symptoms [16].

Multiple moderators were also observed. In this sense, boys with an imprisoned father were more likely to present externalizing problems than girls. In addition, children who lived with their father before incarceration, who had been victims of domestic violence, or whose fathers had not been convicted of a violent crime also showed more externalizing behaviors [8,36].

#### 3.1.5. Internalizing Symptoms

The relationship between parental incarceration and internalizing symptoms in small children was only examined in two studies. One of these studies analyzed paternal incarceration [8], and the other did not differentiate the gender of the incarcerated parent [16]. Only the latter study showed a significant relationship. Concretely, children who experienced parental incarceration had more internalizing symptoms than children who did not [16].

#### 3.1.6. Material Hardship

Two studies were included in this category, one of which focused on paternal incarceration [37], and another which did not differentiate the gender of the parent in prison [11]. Both studies revealed significant results. Parental incarceration was significantly associated with a higher risk of children being homeless [11], and paternal incarceration was related to the lack of material resources [37].

Diverse variables acted as moderators and mediators in the relationship between parental incarceration and material hardship. African American children with a parent in prison had a greater association (*p* < 0.10) with material hardship than other ethnic groups [11]. It was also found that the fact that the father lived with the family before imprisonment was associated with a higher degree of material hardship once the parent was in prison [37]. Finally, an indirect effect of parental imprisonment on material hardship was found through increased economic hardship and reduced institutional support [11].

### 3.2. Children 7 to 11 Years

In this stage, various studies focused on analyzing the association between parental incarceration and physical health, cognitive skills and academic performance, socioemotional skills, delinquent behavior, externalizing and internalizing symptoms, and material hardship.

#### 3.2.1. Physical Health

Four studies were selected for this category, with only two of them distinguishing the gender of the parent in prison [38,39]. Three of these studies found a significant relationship between parental imprisonment and a higher likelihood of presenting health problems. Specifically, a significant relationship was found between paternal incarceration and sleep problems [38], parental incarceration (or just maternal incarceration) and obesity [39], and parental incarceration and higher levels of food insecurity [40]. By contrast, a fourth study showed that parental incarceration did not influence body mass index [41].

Regarding mediating variables, bedtime inconsistency partly mediated the association between paternal incarceration and less sleep duration [38].

#### 3.2.2. Cognitive Skills and Academic Performance

Eight studies were included in this section: seven articles focused on paternal incarceration [6,10,17,34,42,43,44] and only one was on maternal incarceration [18]. A total of four studies found a significant relationship between paternal incarceration and a worse non-cognitive preparation for school entry and a greater possibility of special education attendance in boys [33], worse math problem solving skills, and a greater number of attention and memory problems [6], and, finally, a greater probability of repeating the grade [10], and school suspension [44].

Although four studies did not find a direct association between parental incarceration and cognitive skills and academic performance, some of them found significant moderating and indirect effects [17,42].

Regarding moderating effects, child’s gender seems to play a relevant role. In this respect, girls with a father in prison scored significantly lower in reading comprehension and math problem solving skills than girls who did not have an imprisoned father. In the case of boys, lower levels of memory/attention skills were only found in boys with a parent in prison compared to boys whose father was not incarcerated [6]. Furthermore, paternal incarceration was associated with a greater probability of attending special education in boys, although this effect was not tested in girls [34]. Finally, children with a father in prison and at a low risk level of experiencing parental incarceration were associated with lower reading comprehension, math comprehension, and verbal ability. However, these significant relationships were not found in the group of children with an incarcerated father and a high risk of experiencing parental incarceration [17].

Other variables acted as mediators, such as being less prepared for school in boys (outcome: special education placement [34]), behavior problems and weakened social bonds (outcome: suspension/expulsion from elementary school [44]), supportive maternal caregiving (outcome: reading achievement [42]) and negative perception by teachers (outcome: repeat the grade [10]).

#### 3.2.3. Socioemotional Skills

Only one study entered this category. Paternal incarceration was significantly associated with lower levels of socioemotional skills. This relationship was stronger for those children with a violent father [7].

#### 3.2.4. Delinquent Behavior

It was possible to include four studies in this category. Three of these studies focused on paternal incarceration [17,43,45], and the other one on maternal incarceration [18]. Three of the studies showed a significant relationship between parental incarceration and a greater probability of presenting early juvenile delinquency [18,43,45].

The probability of parental incarceration, categorized in three strata (low, medium, and high), acted as a moderator in two studies, but in different ways. On the one hand, a significant relationship was found between paternal incarceration and a greater probability of presenting early juvenile delinquency in the medium and highest strata but not in the lowest stratum [17]. On the other hand, a significant relationship was observed between maternal incarceration and a greater presence of early juvenile delinquency in the lowest and medium risk strata but not in the highest risk stratum [18]. In addition, having a poor relationship with siblings increased the association between maternal incarceration and greater children’s delinquent involvement [45]. Finally, the association between parental incarceration and a greater presence of early delinquent behavior was significant in boys but not girls [43].

#### 3.2.5. Externalizing Symptoms

Eight studies were incorporated into this category, five of which studied paternal incarceration [17,43,46,47,48], one of which studied maternal incarceration [18], and two which did not distinguish between either type of imprisonment [41,49]. The results of seven of these studies showed a direct, significant, and positive relationship between parental incarceration and these symptoms [17,41,43,46,47,48,49].

Regarding the study that did not find a significant relationship between maternal incarceration and externalizing symptoms [18], it did find a significant association between both variables in those children with a lower probability of experiencing maternal incarceration.

Other variables that also appeared as moderators were empathy and gender. In this sense, the relationship between parental incarceration and aggression problems was no longer significant in children with high empathy levels [49]. On the other hand, the association between paternal incarceration and externalizing symptoms was only significant for boys but not girls [43].

Some mechanisms or mediators were also observed in these studies. Paternal incarceration was associated with higher levels of maternal and child depression, a greater frequency of spanking the child, and less parental involvement, which in turn was associated with a greater presence of externalizing symptoms [46,47].

#### 3.2.6. Internalizing Symptoms

Eight studies were found related to this category. Five of them focused on paternal incarceration [17,43,46,47,48], one on maternal incarceration [18], and two included both types of imprisonment [41,50]. The only study that addressed maternal incarceration did not show a significant relationship with internalizing symptoms [18], while four of the five studies that focused on parental incarceration found a positive association [17,43,47,48].

The risk of suffering paternal and maternal incarceration and gender acted as possible moderators. On the other hand, paternal and maternal incarceration was associated with higher levels of internalizing symptoms in children who had an a priori risk of suffering incarceration of their mother, while this association was not significant in children with a middle or high risk [17,18]. Likewise, paternal incarceration was associated with internalizing problems in boys but not girls [43].

Different mechanisms by which parental incarceration increased internalizing symptoms were also observed. First, maternal incarceration increased maternal depression and stress (mediators), which were associated with higher internalizing symptoms [46]. Second, paternal incarceration was associated with more problems contacting the father in prison, generating more internalizing symptoms in children [50].

#### 3.2.7. Material Hardship

Among the two studies that covered this category, there was a significant association between paternal incarceration and children receiving less financial support from their fathers [51,52]. This relationship was mediated by a decrease in the father’s earnings and the fact that the father in prison and the mother do not live together anymore [51].

### 3.3. Adolescents (12 to 18 Years Old)

Different studies investigated the association between parental incarceration and cognitive skills and school performance, socioemotional skills, risk behaviors, delinquent behaviors, and externalizing and internalizing symptoms during this developmental stage.

#### 3.3.1. Cognitive Skills and Academic Performance

Eight studies were found related to this category. One of these eight studies addressed both paternal and maternal incarceration separately [53], two of them paternal incarceration [15,54], one maternal incarceration [55], and the last three did not discriminate the gender of the parent in prison [56,57,58,59]. The vast majority of these investigations only included variables related to academic performance. Only two studies referred to cognitive skills, specifically attention [15,54].

Five out of eight studies found a significant association between parental incarceration and cognitive skills and academic performance. Having a parent in prison has been associated with poorer school performance (grades), lower educational achievement, higher absenteeism and dropout rates, and more attention problems [53,54,55,57,59]. In contrast with these results, three studies did not find a significant relationship between parental incarceration and academic performance [52,53] and paternal incarceration and attention problems [15].

Significant moderators and mediators were also observed. First, paternal incarceration only during children’s early childhood was significantly associated with more attention problems [54]. Furthermore, parental imprisonment was only related to poor school outcomes among children enrolled in public schools [59].

An indirect relationship was also found between incarceration and poorer school performance and cognitive abilities, through poor quality of the father–child relationship, poorer health, a more negative type of residence and parenting style, and lower economic well-being [54].

#### 3.3.2. Socioemotional Skills

Three studies were included in this category, one regarding paternal incarceration [60] and two that did not differentiate the gender of the parent in prison [61,62]. Only two studies found a significant association between parental incarceration and this category [60,62]. Parental imprisonment was associated with a greater probability of establishing peripheral friendships and maintaining relationships with more conflictive people. However, no significant association was found between parental incarceration and the number of the child’s friends [60,62] or prosocial behavior [61].

It was also found that parental incarceration generated higher levels of caregiving depression which, in turn, generated a worse relationship between the caregiver and the adolescent, concluding in a deterioration of the adolescents’ socioemotional skills (sequential mediation) [61].

#### 3.3.3. Risk Behaviors

Six studies were included in this section, two about the incarceration of the father [63,64] and the rest without differentiating the gender of the parent in prison [14,56,65,66]. The relationship between incarceration and risk behaviors was significant in four studies [12,14,64,65]. Having a father or mother in prison was significantly associated with greater consumption and abuse of substances (alcohol, tobacco, drugs, marijuana, etc.) in two of the four articles that studied this variable [65,66]. Paternal incarceration was also associated with early sexual initiation [64]. Regarding sexual risk, it was only related to parental incarceration in African American children [65]. Likewise, paternal detention, but not incarceration, predicted adolescent alcohol abuse [63].

#### 3.3.4. Delinquent Behavior

Four articles were incorporated into this category, two of which studied paternal incarceration [54,67], and two which did not differentiate the gender of the parent in prison [56,68]. Two of these studies showed a significant relationship between parental imprisonment and delinquent behavior [58,60]. Specifically, an association was found between parental incarceration and increased youth theft [56] and between the incarceration of the father and delinquent behavior [54].

Regarding moderators, paternal incarceration was significantly associated with more delinquent behavior during early childhood only [54]. Concerning mediators, an indirect relationship was also observed between parental incarceration and serious criminal acts through high levels of social disadvantage, poor parental mental health, lower effectiveness in parenting, and a decrease in attachment to fathers [67,68].

#### 3.3.5. Externalizing Symptoms

Ten studies were included in this section. Four of these analyzed the incarceration of the father [15,47,54,63], and the other six the imprisonment of the father or mother without differentiating them [9,12,14,61,68,69]. Six of these studies found that adolescents with an incarcerated parent showed more externalizing symptoms [12,14,47,54,68,69].

To refine these results, moderating and mediating effects were also analyzed. Parental incarceration was a significant predictor of externalizing symptoms in only one of the problematic trajectory groups named “mid-increasing trajectory” (i.e., lower levels of externalizing problems at the age of 10, but with levels gradually increasing to clinically high levels at the age of 16) [9]. The moment of incarceration also moderated the level of externalizing problems. This association was maintained when incarceration occurred during early childhood [54]. Furthermore, children’s closeness with their parents acted as a protective factor against the appearance of these externalizing symptoms [12].

An indirect relationship was also found between parental incarceration and externalizing symptoms mediated by a poor quality father–child and parental relationship, poor health, a more negative type of residence and parenting style, and lower economic well-being [47,54,61].

#### 3.3.6. Internalizing Symptoms

Eleven studies were included in this category. Six of these did not differentiate whether the parent in prison was the father or the mother [12,14,56,69,70,71] while the other five only studied paternal incarceration [15,47,54,63,72]. Five out of the eleven studies revealed a significant relationship between parental incarceration and a greater presence of internalizing symptoms in adolescents [12,14,47,69,72]. These studies included measures of internalizing symptoms such as anxiety, depression, ADHD, suicidal ideation or suicidal or self-injurious behaviors, post-traumatic stress, mental health, and internalizing problems in general.

Several variables were found in this category that exerted a moderating effect as protective variables. Among these were resilience, extracurricular activities, physical activity, quality of sleep [14], and closeness with the father [12]. Screen time also appeared as a risk factor, increasing the likelihood of presenting internalizing symptoms in adolescents with an incarcerated parent. Additionally, there was an indirect effect of parental incarceration on the development of internalizing symptoms in adolescents through caregivers’ depression [71].

### 3.4. Studies That Do Not Differentiate According to Developmental Stage (Children and Adolescents)

Finally, a subsection was created to present the results of all the studies that do not make differences according to age, and that include children and adolescents without distinguishing. These studies introduce measures of both physical and mental health.

#### 3.4.1. Physical Health

Five studies were included in this category, none of which differentiated the gender of the incarcerated parent. Four of them found a significant relationship between parental incarceration and poor physical health [17,73,74,75], while the other one did not find these results [4]. These investigations highlighted parental incarceration as a predictor of various measures related to respiratory, cardiac, bone, muscle, dental, and visual problems, chronic ailments, increased mortality, and unmet needs for medical resources.

Regarding the moderating variables, having health insurance acted as a protective factor in the relationship between parental incarceration and children’s poorer physical health. At the same time, material hardship led to more physical health problems in children and adolescents [73]. Gender also acted as a moderator, with the relationship between parental incarceration and mortality in girls not being significant [74].

#### 3.4.2. Internalizing Symptoms

The relationship between parental incarceration, without differentiating the gender of the parent in prison, and internalizing symptoms in children was studied in three articles. All of them found a significant relationship between parental incarceration and internalizing symptoms [4,5,75]. These studies covered mental health problems, such as Tourette’s syndrome, intellectual disability, learning disabilities, language problems, ASD, developmental delay, anxiety, depression, and ADHD.

A summary of the direct effects of parental imprisonment on different outcomes, as well as the effects of moderators and mediators are included in Table 1 and Table 2. Additionally, the results of each study included in this review may be consulted in Appendix A (Table A1, Table A2, Table A3 and Table A4).

**Table 1:** Summary of significant direct effects of parental incarceration on the different outcomes.

	Children 0 to 6 Years	Children 7 to 11 Years	Adolescents (12 to 18 Years)	Developmental Stage Is Not Determined
Physical health	3/4 (75%)	3*/4 (75%)	-	4/5 (80%)
Cognitive skills and academic performance	4/5 (80%)	4/8 (50%)	5/8 (62.5%)	-
Socioemotional skills	0/1 (0%)	1/1 (100%)	2/3 (66.6%)	-
Risk behaviors	-	-	4/6 (66.6%)	-
Delinquent behaviors	-	3/4 (75%)	2/4 (50%)	-
Externalizing symptoms	2/3 (66.6%)	7/8 (87.5%)	6/10 (60%)	-
Internalizing symptoms	1/2 (50%)	6/8 (75%)	5/11 (45.5%)	3/3 (100%)
Materials hardship	2/2 (100%)	2/2 (100%)	-	-

* Note: The direction of the relationship of one of these studies [39] is contrary to the rest of the evidence (maternal incarceration–higher physical health).

**Table 2:** Summary of moderators and mediators in the relationship between parental incarceration and different outcomes.

	**Children 0 to 6 Years**		**Children 7 to 11 Years**		**Adolescents (12–18 Years)**		**Developmental Stage Is Not Determined**	
	**Moderation**	**Mediation**	**Moderation**	**Mediation**	**Moderation**	**Mediation**	**Moderation**	**Mediation**
Physical health	Father in prison who exerts domestic violence (*p* < 0.10)	-	-	Bedtime consistency	-	-	Child’s gender, household material hardship, child does not have medical insurance.	-
Cognitive skills and academic performance	Child’s gender	Teacher’s perceptions	Child’s gender, risk of parental incarceration	School readiness, maternal care, behavioral problems, weak social relationships and teacher´s perceptions	Moment of incarceration, school setting	Quality of the parent–child relationship, health, type of residence, parenting style, economic well-being	-	-
Socioemotional skills	-	-	-	Father in prison who exerts violence	-	Caregiver depression, quality of the caregiver–child relationship	-	-
Risk behaviors	-	-	-	-	Child’s gender, children’s sleep quality, father in prison lived with the child before incarceration.	Externalizing problems	-	-
Delinquent behaviors	-	-	Child’s gender, risk of parental incarceration, negative sibling relationship quality	-	Moment of incarceration	Social disadvantages, parents´ mental health, parenting effectiveness, the reduction of attachment to fathers	-	-
Externalizing symptoms	Child’s gender, father in prison lived with the child before incarceration, father in prison exerted domestic violence, crime for which the parent was arrested	-	Child’s gender, empathy, risk of parental incarceration	Maternal depression, frequency of spankings, parental implication, child depression	Closeness to father, moment of incarceration, previous externalizing problems, resilience, extracurricular activity	Caregiver depression, caregiver–child relationship, adolescent depressive symptoms, perceived social support, parenting style, change of residence, economic well-being	-	-
Internalizing symptoms	-	-	Child’s gender, risk of parental incarceration	Maternal depression and stress, problems experienced trying to contact the parent in prison	Resilience, extracurricular activity, physical activity, sleep quality, screen time, closeness to incarcerated parent	Caregiver depression	-	-
Material hardship	Ethnic group (*p* < 0.10), cohabitation of the father with the family prior to incarceration	Family economic difficulties and reduced institutional support	-	Decrease in father’s earnings, incarcerated father and mother do not live together	-	-	-	-

## 4. Discussion

During childhood and adolescence, a significant percentage of children are exposed to parental incarceration. This phenomenon may have a negative impact on the well-being and development of children and adolescents [4]. This systematic review has focused on studies examining the differences in the effect of parental incarceration on the well-being and development of children across three different developmental stages: children aged 0 to 6 years, children aged 7 to 11 years, and adolescents aged 12 to 18 years. A fourth group of studies that did not differentiate the age of the children was also included. To this end, studies were selected following a series of inclusion and exclusion criteria, highlighting the presence of a comparison group, control variables, and a quantitative approach, and were categorized based on the developmental stage of the children. This distinction allowed us to observe if there were variations in the results depending on the children’s stage of development. The second goal of this systematic review was to address the analysis of moderating and mediating variables in the relationship between parental incarceration and the children’s development and well-being also considering the above-mentioned three different developmental stages.

### 4.1. Parental Incarceration and Children’s Development and Well-Being

There is strong evidence to show that parental incarceration has a significant impact on the well-being and development of children and adolescents. This has been observed across all relevant outcomes related to children’s well-being and development, except for socioemotional skills in children from 0 to 6 years old (only one study was found for this outcome, which does not represent sufficient empirical evidence). These results are consistent with findings in previous systematic reviews that have also found a general negative effect of parental incarceration on the well-being and development of these children [21,22,28].

However, this present review finds a more specific and unique pattern of significant associations between parental incarceration and a worse state of children’s well-being and development in different developmental stages. This is determined by selecting outcomes with higher significance (categories of outcomes with at least four studies, and 75% of them showed significant negative effects of parental incarceration). In this regard, in children aged 0 to 6 years, parental incarceration had adverse effects on cognitive skills and academic performance (80%) and physical health (75%). In children aged 7 to 11 years, it had adverse effects on externalizing symptoms (87.5%) internalizing symptoms (87.5%), and delinquent behaviors (75%). In adolescence, the adverse effects of parental incarceration were less pronounced and only appeared on risk behaviors (66.6%), cognitive skills in academic performance (62.5%), and externalizing symptoms (60%) when 60% was selected as the cut-off point. It is worth noting that some outcomes, such as material hardship or socioemotional skills were not included in the analysis due to a lack of sufficient studies.

In general, we can observe that from 0 to 6 years there was a particularly important effect on cognitive and educational aspects. More attentional problems [8], worse preparation for school [34,35], and a greater probability of repeating grades [10] were found in children with an incarcerated parent. This stage is especially important for cognitive development, especially for the start of the development of the attentional filter [76], and all this influences better academic adaptation to preschool and kindergarten [77].

Considering that the first years of a child’s life are key for the acquisition of literacy and initial math skills, it is especially relevant that most problems occur in this stage as this can mean difficulties that can be carried over throughout children’s education.

While the review by Poehlmann-Tynan and Turney [22] places attentional problems between 3 and 5 years and between 9 and 16 years, and school difficulties between 6 and 17 years, and the review by Luk et al. [28] does not clearly specify the age (0 to 17 years), our review highlights these problems which appear in the studies with a higher percentage from 0 to 6 years. However, these difficulties appear in all the stages studied to varying degrees.

On the other hand, worse physical health also appears as one of the most relevant results in the 0 to 6 years stage, more so than in later stages. Previous reviews have also found a relationship between parental incarceration and physical health [26,78,79,80], even though only some of them break their results down by the developmental stage [21,22]. In this sense, Poehlmann-Tynan and Turney [22] place adverse health effects at birth from 9 to 16 years old, and Austin et al. [21] in infants (11 out of 10 studies) and from early childhood to late adolescence (7 out of 10 studies). Although our review also finds an important effect between parental incarceration and physical health in the block of studies which did not determine the developmental stage, the most apparent evidence of adverse effects appears in the 0 to 6 years stage. This result partially coincides with Austin et al. [21], since these authors also observed a higher incidence at birth. However, they include a too-broad developmental stage from early childhood to adolescence.

There is no evidence of whether such manifestations express their emotional distress, either because of how children express themselves during this developmental stage or because adults do not often identify forms of emotional expression that do not follow the usual adult rules.

Regarding the 7–11 years old stage, the highest percentage of studies with a significant association was found in children exhibiting externalizing symptoms, followed by delinquent behavior and internalizing symptoms. In this developmental stage, the children’s behavior seems to play a crucial role, as parental incarceration negatively affects externalizing and delinquent behavior. At this life stage, children are expected to have gained self-control [81] to regulate their behavior, but it seems that the absence of a parent at home may disrupt this process. Additionally, cognitive development at this life stage makes these children and their peers more aware of the parent’s situation than in early childhood, which may increase feelings of stigma and internalizing symptoms.

Again, some previous reviews have found an association between parental incarceration and experiencing externalizing and internalizing behavior problems at various points across childhood, but without specifying the specific stages [78]. While there is a general agreement on the presence of externalizing behavior problems [25], there are conflicting results regarding the significant association between mental health problems and/or internalizing behavior symptoms. Some studies affirm this association [22,28,78,79], while others do not [25]. According to Poehlmann-Tynan and Turney’s [22] and Wildeman et al.’s [80] reviews, the consequences of parental incarceration appear in earlier developmental stages in the case of externalizing behavioral problems and later for internalizing behavioral problems. In particular, Poehlmann-Tynan and Turney’s [22] review states that whereas more attention problems and aggression appear between 3 and 5 years old, externalizing and internalizing problems, as well as antisocial behaviors and delinquency, appear in middle childhood. The current review is consistent with the findings of Poehlmann-Tynan and Turney [22], as it also found that children exhibit externalizing, internalizing, and early delinquent behavior problems in middle childhood. However, this review makes a novel contribution by finding that a larger number of studies have identified children experiencing externalizing and internalizing behavior problems at 7 to 11 years old than in other developmental stages.

Additionally, when analyzing each developmental stage individually, we find that the most robust results correspond to children 7 to 11 years of age. This original contribution of the current review could be explained because the transition to adolescence is included in this developmental stage (the mean age of most of these studies is around 9 years old, and this transition is a challenging moment in a child’s life [82]). In other words, we speculate that the effect of parental incarceration on children’s development and well-being might be more pronounced at this developmental stage.

Although few studies have investigated the material hardship experienced by these families, it is important to note that parental incarceration has been significantly associated with higher levels of this variable in all studies conducted, both in early and middle childhood.

Regarding adolescents, when reducing to 60% significant studies (with a minimum of four studies for each outcome), risk behaviors, cognitive skills, academic performance, and externalizing symptoms were found to be the most relevant adverse effects of parental incarceration.

Health risk behaviors mainly comprised substance abuse and sexual behavior. With regard to adolescent sexual behavior, previous studies have also shown a link to the early onset of sexual relationships and sexual risks [28,79]. In addition, previous reviews have also found a significant association between parental incarceration and substance abuse (alcohol, tobacco, drugs, marijuana, etc.) [26,28,78,79,80], although one review did not find a significant association specifically with illicit drugs [25]. These two types of risk behaviors are typically observed from adolescence onwards [83,84], and their association with parental incarceration during this developmental stage was found to be statistically significant in three out of four (75%) studies included in this category.

As was the case in the 7 to 11 years old stage, parental incarceration was also associated with externalizing symptoms in adolescence. As previously mentioned, different reviews have found this significant association, although most of them have not specified a concrete age group. At this developmental stage, Poehlmann-Tynan and Turney’s [22] and Luk et al.’s reviews [28] make references to the association between parental incarceration and externalizing symptoms. However, as also described above, more studies have found a significant relationship between these two variables in middle childhood than in adolescence. At this developmental stage, human beings progressively gain autonomy and the ability to elaborate and express what is happening to them cognitively, so children’s discomfort and disruptive behaviors become more visible than in the previous stage. At the same time, these behaviors, such as early delinquent behaviors, are not usually attended to or considered especially problematic by adults until adolescence. Unfortunately, this consideration often hides the need for early and preventive intervention that may be more effective.

Finally, having an incarcerated parent has also been associated with lower grades and achievement, higher absenteeism and dropout rates, and more attention problems [61,62,63,65,67] in adolescence. Previous reviews have also found a relationship between parental incarceration and different school-related problems without clearly specifying the age group [25,78,80], or placing this relationship in different developmental stages [22,28]. This current review finds evidence of this relationship in all developmental groups, but the number of studies that found this relationship in adolescence and early childhood is lower compared to middle childhood.

In general, this review has found a lower percentage of studies with significant results in adolescence. This fact may be due to adolescents’ responses being more likely to come from the adolescents themselves rather than from their primary caregiver, as is the case in earlier developmental stages. Future studies should control the effect of the respondent on the association between parental incarceration and different outcomes.

### 4.2. Moderating and Mediating Factors Depending on Children’s Developmental

Furthermore, the second research question addressed the possible influence of moderating and mediating variables on the relationship between parental incarceration and the measures related to the development and well-being of children. Previous reviews have also attempted to examine the impact other factors have on children with an incarcerated parent, but either they did not specifically study the mediating or moderating role [28] or were not comprehensive in describing all possible moderating or mediating effects [22,56]. Our results identify some moderating and mediating effect patterns within each age group. However, finding a clear pattern is challenging due to the limited number of studies addressing these issues.

Among the moderating variables, gender stands out for its importance. Boys often reported more significant problems compared to girls in different categories, such as physical health, cognitive abilities and academic performance, externalizing problems, juvenile delinquency, or risk behaviors, e.g., [8,34,36,43,64]. The relationship is also moderated by the children’s risk of parental incarceration [17], the timing of the incarceration in the child’s life [54], and whether the father lived with the child before being incarcerated [8]. In addition, some characteristics of these children, such as empathy or resilience, act as protective factors, reducing the impact of incarceration in children with higher levels of these abilities, e.g., [14,49]. Some variables such as gender, exposure to incarceration-related events, or timing of incarceration also showed this effect in other reviews [28,56].

Gender socialization could influence this issue. Gender stereotypes emphasize different characteristics for boys and girls: initiative, emotional and physical strength, limited emotional expression of fragility or vulnerability in boys, and tenderness, care, and the expression of emotional discomfort and fragility in girls.

Regarding mediating variables, the mental health of both child and caregiver [47,61], the quality of the child–caregiver relationship [61], the level of parental involvement, and the type of parenting model employed by the parents [68] are of particular importance. In this sense, parental incarceration is associated with worse children’s mental health, a weaker child–caregiver relationship, reduced parental involvement, and a more negligent parenting style. These factors, in turn, are associated with lower levels of variables that describe the children’s well-being and development. Consistent with our results, previous reviews also highlighted various family aspects as key mediators between parental incarceration and different outcomes [28].

Concerning developmental differences, it is not easy to make a synthesis. As mentioned, each author decided to study certain variables as moderators and mediators, and the same variables may not have been studied for each stage.

Despite this, it has been possible to observe that, in the case of moderators, most of them are common across the three developmental stages. In this sense, the child’s gender, whether the father lived with the child before incarceration, and the timing of the incarceration, act as moderators regardless of the child’s age. Other moderators only appear in adolescence, such as the relationship with the father and different variables related to the adolescent’s extracurricular activities.

The effects of mediating variables have hardly been studied for the youngest children, so it is not possible to compare them with the other two developmental stages. In the case of middle childhood and adolescence, it is observed that the effect of caregiver’s health is the most important, and is present in both stages. Caring for the caregiver promotes the development and well-being of children and adolescents. Similarly, the child’s health and behavioral problems, as well as socio-economic characteristics, also stand out as mediators in both stages. On the other hand, in the case of adolescents, the quality of family relationships (between parents and the child) and the parenting style are highly important as mediating variables, not appearing in other stages. Moreover, the perception of teachers appears during early childhood and middle childhood, probably as a result of the stigma that accompanies these children for having a parent in prison.

### 4.3. Practical and Theoretical and Practical Implications

This review brings up important practical and theoretical implications. The evidence suggests that parental incarceration adversely affects children’s development and well-being. These results highlight the need for intervention with these children and their families at a medical, psychological, social, political, and academic level. Policies and strategies for prevention and correction should be also taken into consideration.

From a developmental perspective, although all the outcomes studied in this review indeed deserve attention at all developmental stages, the results obtained highlight greater evidence that certain stages should be considered for any type of intervention. In this sense, work on physical health, cognitive skills, and academic performance is especially important in early childhood. Interventions should explore how these aspects may also express the child’s distress. Working with caregivers on identifying the child’s actual mood and improving effective communication will help prevent future problems in the child. In addition, internalizing and externalizing symptoms and the early delinquent behaviors appear in middle childhood, while risk behaviors (66.6%), cognitive skills and academic performance (62.5%), and externalizing symptoms (60%) appear in adolescence. Although there are few studies on the material hardship suffered by these families, it is also important to palliate the effects that this has since in all the studies it is associated with parental incarceration.

On the other hand, it seems important to place special emphasis on middle childhood, as this is the stage in which most studies show significant negative effects of parental incarceration. Interventions at this stage can help compensate for problems stemming from early childhood and prevent future problems in adolescence.

As far as psychological intervention is concerned, several programs have been implemented so far with both parents and children to support them in the situation of having an incarcerated parent [85,86,87]. However, thanks to the analysis by developmental stages, it is possible to determine beforehand the areas in which they may face more difficulties, allowing for more directed interventions.

Additionally, the analysis of mediating and moderating variables based on the developmental stage offers useful information for intervention. For example, being a male child was a frequently observed risk factor, so this group should be a reference for intervention. Moreover, it has been observed that the caregiver’s health, as well as the relationship between the caregiver and the child, is crucial in the impact on the well-being and development of the child, especially in middle childhood and adolescence. For this reason, programs should not only be oriented towards working with minors but also with the caregiver figure, focusing on enhancing their well-being and creating a good relationship between the caregiver and the minor [88]. In this sense, an appropriate intervention could be systemic family therapy, since much importance is given to the minor’s interpersonal relationships with different agents who influence their development (mainly the primary caregiver and the parent in prison). This type of intervention has already been put into practice with children of prisoners, producing positive results [89].

In terms of theoretical contributions, the results of this review largely coincide with the theoretical model developed by Austin [21] to explain the physical health of these children. This model, which explains parental-incarceration-related intergenerational and chronic stress, integrates the most relevant theoretical models in this field of study [90,91,92,93,94,95]. This model mainly indicates that parental incarceration has an influence on physical health throughout the family (material resources, family relationships, etc.) and the child who is also in the family (e.g., stigma, internalizing/externalizing symptoms, etc.). Conversely, certain characteristics act as moderators (e.g., parent and child gender). On the other hand, racial and socioeconomic disadvantages reinforce the effect of all these factors. In view of our review, the application of this model to explain children’s development and well-being would replace physical health with the different outcomes that we have reviewed in this work. Notably, the mental health of the caregiver and his relationship with the child would be within the family variables that act as mediators, and the child’s gender would be a moderator variable. To these, the moderators and mediators included in Table 2 would be added. A clear contribution of our work would be to place this model on a chronological axis, as the Bronfenbrenner ecological model [90] does, considering the developmental stage in which the child is found, as that can determine different outcomes, mediators, and moderators.

### 4.4. Limitations and Future Lines of Research

The different number of studies regarding each developmental stage and each outcome may be a confounding factor in this review. The varying number of studies for each developmental stage and outcome may pose a challenge in this review. For instance, the number of studies for the 0-to-6-years-old developmental stage is considerably lower than for the rest of the stages, except in the categories of physical health and cognitive abilities, and academic performance. This may lead to less accurate results for this group than for other age groups and emphasizes the need for future research to provide more scientific evidence. We have purposely focused on the outcomes with more empirical evidence, especially in the discussion. For this reason, some outcomes may be too underrepresented to achieve a better description of the findings with more empirical support.

Furthermore, the gender of the parent in prison has also been considered, noting that most studies only considered parental incarceration or did not distinguish between the gender of the parent in prison. It is probable that in the latter case, a greater number of men than women were included due to the higher ratio of men in prison [28]. Only two studies in this review analyzed exclusively maternal incarceration, with only one showing significant results [55]. The lack of evidence makes it impossible to determine whether maternal incarceration is less harmful than paternal incarceration. It is necessary to continue investigating the impact of maternal incarceration on children and adolescents.

Additionally, most of the studies included in this review have been carried out in the United States, which makes comparison with other countries difficult due to cultural differences in the characteristics of the population and the prison system. Cross-cultural studies would be necessary to obtain more representative and comparable results. Additionally, some of the studies used in this review were obtained from two specific databases: “The Fragile Families and Child Wellbeing Study” and “The National Longitudinal Study of Adolescent Health,” which may bias the results obtained. These national databases work with many participants, whereas other studies have used short samples. Differences in the sample sizes also present a limitation in representativeness and statistical significance.

## 5. Conclusions

This work contributes to previous reviews in this field by offering a developmental view of the effects of parental incarceration and the moderating and mediating variables. In this sense, greater evidence of the association between parental incarceration and poorer physical health and cognitive skills and academic performance in early childhood (0–6 years old) has been found, with a higher presence of externalizing and internalizing symptoms and early delinquent behaviors in middle childhood (7–11 years old), and finally, a higher presence of risk behaviors, externalizing symptoms, and lower cognitive skills and academic performance in adolescence (12–18). Additionally, middle childhood presents a greater number of studies in which the association between parental incarceration and different outcomes is significant. Finally, being a male child appears as the moderating risk factor with the most evidence, especially in the three developmental stages analyzed, although with a greater presence in middle childhood, while the mental health of the primary caregiver and the quality of their relationship with the child are the main mediating variables that appear both in middle childhood and adolescence.

To conclude, there is a need for further evidence on the impact of parental incarceration and the mediating and moderating factors from a developmental perspective, emphasizing cultural differences [3,28]. The importance of working on the design, implementation, and evaluation of interventions with a systemic approach aimed at this population group must not be forgotten.

## Data Availability

Not applicable.

## Appendix A

**Table A1:** Summary of studies included in the systematic review. Children aged 0 to 6 years old (early childhood).

Criterion Variable	Study	Characteristics of the Study:		Results
		(a) Size (b) Mean Age (c) Parent in Prison	(d) Type of Study (e) Control Variables	
Physical health: General health assessment	Geller et al. (2012) ^a^ [8]	(a) N = ~3000 (b) M = 5 years ^b^ (c) PP = F	(d) TS = Long. (e) CV ≥ 20	- Parental incarceration was not related to child health measures.
Physical health: Sleep and eating behaviors	Jackson and Vaughn (2017) [31]	(a) N = 2100–2388 (b) M = 5 years ^b^ (c) PP = B	(d) TS = Long. (e) CV = 5–10	- Parental incarceration was significantly associated with eating and sleeping problems. Single-parent incarceration predicted all measures except fast food consumption. Similarly, when both parents were incarcerated, the relationship was significant for all measures except fast food and starch consumption. - When differentiating between maternal and paternal incarceration, it was found that the relationship between maternal incarceration and sleep-related problems and starch consumption ceased to be significant. This is also the case for the relationship between paternal incarceration and the consumption of junk food.
Physical health: Food insecurity	Turney (2015) [33]	(a) N = 3004 (b) M = 5 years ^b^ (c) PP = F	(d) TS = Long. (e) CV ≥ 20	- For those children living with their fathers prior to incarceration, paternal imprisonment was significantly associated with an increased risk of current food insecurity and an increased risk of developing food insecurity in the future. The results were not significant for children who did not live with their fathers prior to incarceration.
Physical health: Infant mortality	Wildeman (2012) [32]	(a) N = 42,544 (b) M = 0–19 months (c) PP = B	(d) TS = Cross. (e) CV ≥ 20	- Parental incarceration predicted an increase in infant mortality. - Moderation: The findings show a statistical trend (*p* < 0.10) that this association is concentrated mostly in children whose fathers did not report domestic violence.
Cognitive skills and academic performance: Academic performance	Casey et al. (2015)^a^ [16]	(a) N = 138 (b) M = 5.75 years (c) PP = B	(d) TS = Cross. (e) CV ≤ 5	- No significant relationship was found between having experienced parental incarceration and poorer academic performance.
Cognitive skills and academic performance: Attention difficulties and verbal skills	Geller et al. (2012) ^a^ [8]	(a) N = ~3000 (b) M = 5 years ^b ^ (c) PP = F	(d) TS = Long. (e) CV ≥ 20	- Recent incarceration of fathers was significantly associated with increased attention problems in children, but a within-individual change in attention problems is not significant.
Cognitive skills and academic performance: School readiness (cognitive and non-cognitive)	Haskins (2014) ^a^ [34]	(a) N = 4311 (b) M = 5 years ^b^ (c) PP = F	(d) TS = Long. (e) CV ≥ 20	- Moderation (separation of groups without a proper moderation test): Parental incarceration was significantly associated with poorer non-cognitive readiness for school entry in boys but not in girls. The differences were not significant for cognitive readiness.
Cognitive skills and academic performance: School readiness	Testa and Jackson (2021) [35]	(a) N = 15,402 (b) M = 3.99 years (c) PP = B	(d) TS = Cross. (e) CV = 20	- Concerning the four categories that reflect school readiness (early learning skills, self-regulation, social-emotional development, physical health, and motor development), children exposed to parental incarceration are at much higher risk of not achieving “on-track” status.
Cognitive skills and academic performance: Early grade retention	Turney and Haskins (2014) ^a^ [10]	(a) N = 947 (b) M = 9 years ^b^ (asked about their 0–6-years-old) (c) PP = F	(d) TS = Long. (e) CV ≥ 20	- Children who experienced paternal incarceration for the first time between the ages of 0 and 5 were more likely to repeat a grade in early childhood education. - There is no evidence of variation regarding gender, ethnicity, or whether or not the father had resided with the children prior to incarceration. - Mediation: When controlling for the different mechanisms through which parental incarceration might influence children’s likelihood of repeating a grade, most of the relationship was due to the teachers’ perceptions, which is a mediating variable.
Socioemotional skills: Emotion recognition	Hindt et al. (2016) [20]	(a) N = 128 (b) M = 5.23 years (c) PP = B	(d) TS = Cross. (e) CV = 5–10	- Although children with incarcerated parents showed significantly fewer positive emotions than the comparison group, the relationship between parental incarceration and poorer emotion recognition was not significant. On the other hand, children with incarcerated parents showed negative biases (a tendency to interpret the neutral/positive more negatively) compared to those without incarcerated parents.
Externalizing symptoms: Externalizing symptoms	Casey et al. (2015) ^a^ [16]	(a) N = 138 (b) M = 5.75 years (c) PP = B	(d) TS = Cross. (e) CV ≤ 5	- No significant relationship was observed between parental incarceration and exhibiting more externalizing symptoms.
Externalizing symptoms: Externalizing symptoms	Geller et al. (2012) ^a^ [8]	(a) N = ~3000 (b) M = 5 years ^b^ (c) PP = F	(d) TS = Long. (e) CV ≥ 20	- Children of incarcerated parents displayed more aggressive behaviors than their peers without imprisoned parents. These results held, and even increased slightly, when considering whether the incarceration was recent. - Moderation: Several moderating variables were observed, such as having lived with the father before incarceration, with these children presenting more problems than those who had not lived with the father. In addition, having suffered domestic violence was associated with a reduction of externalizing problems. Gender also moderated the relationship, as girls’ results were no longer significant.
Externalizing symptoms: Physical, aggressive behaviors	Wildeman (2010) [36]	(a) N = 2275 (b) M = 5 years ^b^ (c) PP = F	(d) TS = Long. (e) CV = 15–20	- The results showed a significant relationship between parental incarceration and higher levels of physical aggression in boys with incarcerated fathers. - Moderation (separation of groups without a proper test of moderation): Gender acted as a moderator of the relationship, observing that, in girls, paternal imprisonment did not increase physical aggression, and it could even be a protective factor, although the results are not robust. - Moderation: The association of paternal incarceration with physical aggression of children focused on those whose fathers had not been arrested for violent crimes or had not been abusive to the children’s mother.
Internalizing symptoms: Internalizing symptoms	Casey et al. (2015) ^a^ [16]	(a) N = 138 (b) M = 5.75 years (c) PP = B	(d) TS = Cross. (e) CV ≤ 5	- Children who experienced parental incarceration had more internalizing symptoms than children without an incarcerated parent.
Internalizing symptoms: Internalizing symptoms	Geller et al. (2012) ^a^ [8]	(a) N = ~3000 (b) M = 5 years ^b^ (c) PP = F	(d) TS = Long. (e) CV ≥ 20	- Parental incarceration did not significantly predict internalizing symptoms in children of prisoners, compared with other children who had not experienced parental incarceration.
Material hardship: Material hardship	Schwartz-Soicher et al. (2011) [37]	(a) N = 3834 (b) M = 5 years ^b^ (c) PP = B	(d) TS = Long. (e) CV ≥ 20	- The results showed a relationship between paternal incarceration and family material hardship (increased financial and other family strains). - Moderation: The hardship’s effect of paternal incarceration was more pronounced for families that cohabited with the father prior to his incarceration.
Material hardship: Child homelessness	Wildeman (2014) [11]	(a) N = 3774 (b) M = 5 years ^b^ (c) PP = B	(d) TS = Long. (e) CV ≥ 20	- Recent paternal but not maternal incarceration substantially increased the risk of child homelessness. - After selecting an appropriate comparison group, the risk of child homelessness increased by 2–4% in the group with an incarcerated father. - Moderation: A statistical trend (*p* < 0.10) pointed out that these effects intensified among African American children. - Mediation: Part of the relationship was mediated by increased family economic difficulties and decreased access to institutional support.

N = Size; M = Mean Age; PP = Parent in Prison; TS = Type of Study; CV = Number of Control Variables; F = Father; M = Mother; B = Both. Cross. = Cross Sectional; Long. = Longitudinal^; a^ Studies that include different types of criterion variables or focus on more than one developmental stage.; ^b^ Mean age is not shown, only the age of the children at the time of the assessment.

**Table A2:** Summary of studies included in the systematic review. Children aged 7 to 11 years old (middle childhood).

Criterion Variable	Study	Characteristics of the Study:		Results
		(a) Size (b) Mean Age (c) Parent in Prison	(d) Type of Study (e) Control Variables	
Physical health: Sleep schedules and sleep regulation	Branigan and Meyer (2020) [38]	(a) N = 3246 (b) M = 9 years ^b^ (c) PP = B	(d) TS = Long. (e) CV = 10–15	- Paternal, but not maternal, incarceration was significantly associated with less effective sleep regulation, including less consistent bedtime. There was also a difference in mean daily sleep time compared to children who had not experienced parental incarceration. - Mediation: Bedtime consistency partly mediated the association between paternal incarceration and total sleep duration.
Physical health: Overweight	Branigan and Wildeman (2019) [39]	(a) N = 2141 (b) M = 9 years^b^ (c) PP = B	(d) TS = Long. (e) CV = 15–20	- Maternal, or parental, incarceration was associated with being overweight or obesity in children. In the case of paternal incarceration, the results were not significant.
Physical health: Food insecurity	Cox and Wallace (2016) [40]	(a) N = 2849 (b) M = 9 years ^b^ (c) PP = B	(d) TS = Long. (e) CV ≥ 20	- Parental incarceration increased the likelihood of food insecurity by about 4%.
Physical health: Body mass index	Haskins and McCauley (2019) ^a^ [41]	(a) N = 1664 (b) M = 9 years ^b^ (c) PP = B	(d) TS = Long. (e) CV = 5–10	- No significant relationship was found between parental incarceration and body mass index.
Cognitive skills and academic performance: Reading skills	Bridgewater and Yates (2021) [42]	(a) N = 180 (b) M = 10 years ^b^ (c) PP = F	(d) TS = Long. (e) CV = 5–10	- This study did not show a significant negative effect of parental incarceration on children’s reading skills. - Mediation: A significant indirect relationship was observed through the mediation of the mother’s care. - Moderator: This relationship was moderated by gender and was no longer significant for girls when gender was included.
Cognitive skills and academic performance: Special education attendance	Haskins (2014) ^a^ [34]	(a) N = 4311 (b) M = 5 years ^b^ (c) PP = F	(d) TS = Long. (e) CV ≥ 20	- Moderation: Paternal incarceration predicted a greater likelihood of children (boys) attending special education than children who had not been exposed to their father’s incarceration. This effect was not found for girls. - Mediation: School readiness measured four years earlier mediated the relationship between paternal incarceration and attending special education.
Cognitive skills and academic performance: Task completion	Haskins (2015) ^a^ [43]	(a) N = 2150 (b) M = 9 years ^b^ (c) PP = F	(d) TS = Long. (e) CV ≥ 20	- Paternal incarceration did not influence task completion in children exposed to this phenomenon, compared to the comparison group. There was also no significant effect when dividing children according to gender.
Cognitive skills and academic performance: Reading and mathematical comprehension, vocabulary, and memory/attention	Haskins (2016) [6]	(a) N = 2192 (b) M = 9.29 years (c) PP = F	(d) TS = Long. (e) CV ≥ 20	- Father’s incarceration was associated with deficits in the dimensions of mathematical resolution and children’s memory and attention skills. However, the results of vocabulary skills and reading comprehension were not significant. - Moderation: In the case of girls with a parent in prison, mathematical comprehension scores were significantly lower than those of their peers who had not experienced parental imprisonment. In the case of boys with a parent in prison, differences were only significant in memory/attention skills.
Cognitive skills and academic performance: School punishment/expulsion	Jacobsen (2019) [44]	(a) N = 3201 (b) M = 9 years ^b^ (c) PP = F	(d) TS = Long. (e) CV ≥ 20	- Children whose fathers were incarcerated before first grade were more likely to be suspended or expelled by age nine than other children. A total of 16% of children whose fathers were in prison were suspended/expelled from school compared to 9% of other children. This result was limited to children who lived with their fathers prior to incarceration. - Mediation: The association between paternal incarceration and suspension/expulsion from elementary school was partially explained by behavioral problems and weakened social bonds.
Cognitive skills and academic performance: Reading and mathematical comprehension, and verbal skills	Turney (2017) ^a^ [17]	(a) N = 3065 (b) M = 9 years ^b^ (c) PP = F	(d) TS = Long. (e) CV ≥ 20	- No significant relationship was found between parental incarceration and the different measures of cognitive skills (using matched estimates based on propensity scores). - Moderation (separation of groups without a proper test of moderation): When parents were divided into three groups with different probability of risk of entering prison (low, medium, and high), a significant relationship was observed between parental incarceration and lower reading comprehension in the lowest and medium strata, but not in the highest stratum. Additionally, a significant relationship was found between parental incarceration and lower mathematical comprehension and verbal skills in the lowest-risk stratum but not in the medium and highest-risk strata.
Cognitive skills and academic performance: Early grade retention	Turney and Haskins (2014)^a^ [10]	(a) N = 947 (b) M = 9 years ^b^ (c) PP = F	(d) TS = Long. (e) CV ≥ 20	- Children of parents who had been incarcerated for the first time when the child was between one and five years were more likely to repeat a grade in primary school. - There was no evidence of variation concerning gender, ethnicity, or whether or not the father had previously resided with the children. - Mediation: Most of the relationship was mediated by the teachers’ perceptions.
Cognitive skills and academic performance: Verbal skills	Turney and Wildeman (2015) ^a^ [18]	(a) N = 3197 (b) M = 9 years ^b^ (c) PP = M	(d) TS = Long. (e) CV ≥ 20	- No significant relationship was found between maternal incarceration and verbal aptitude scores in their children.
Socioemotional skills: Socioemotional skills	Washington (2018) [7]	(a) N = 3225 children b) M = 9 years ^b^ (c) PP = F	(d) TS = Long. (e) CV = 10–15	- A significant association between paternal incarceration and poorer socioemotional functioning in nine-year-old children was found. - Moderation: Violent father behavior moderated the relationship between paternal incarceration and teacher-reported poorer socioemotional functioning. The children of more violent fathers showed more problems.
Delinquent behaviors: Delinquent behaviors	Haskins (2015) ^a^ [43]	(a) N = 2150 (b) M = 9 years ^b^ (c) PP = F	(d) TS = Long. (e) CV ≥ 20	- Paternal incarceration significantly predicted delinquent behavior in children exposed to it compared to children who had not experienced it. - Moderation: Considering the gender of the children, the results remained significant for boys but not for girls.
Delinquent behaviors: Delinquent behaviors	Turney (2017) ^a^ [17]	(a) N = 3065 (b) M = 9 years ^b^ (c) PP = F	(d) TS = Long. (e) CV ≥ 20	- There was a significant relationship between parental incarceration and more delinquent behaviors in children. - Moderation (separation of groups without a proper test of moderation): When fathers were divided into three groups with different probability of risk of entering prison (low, medium, and high), a significant relationship was observed between paternal incarceration and a higher probability of presenting early juvenile delinquency in the medium and highest strata, but not in the lowest stratum.
Delinquent behaviors: Juvenile delinquency	Turney and Wildeman (2015) ^a^ [18]	(a) N = 3197 (b) M = 9 years ^b^ (c) PP = M	(d) TS = Long. (e) CV ≥ 20	- Maternal incarceration was not significantly associated with children’s delinquent behaviors - Moderation (separation of groups without a proper test of moderation): When parents were divided into three groups with different probability of risk of entering prison (low, medium, and high), a significant relationship was observed between maternal incarceration and a higher presence of early juvenile delinquency in the lowest and medium risk strata but not in the highest risk stratum.
Delinquent behaviors: Delinquency	Woodard and Copp (2016) [45]	(a) N = 3391 (b) M = 9.28 years (c) PP = B	(d) TS = Long. (e) CV = 10–15	- Maternal incarceration was significantly associated with higher delinquency levels at age nine. In addition, when considering the previous incarceration of the father, the results were also significant. - Moderation: Regarding the child’s gender, the results were higher for boys than for girls. The child’s relationship with their siblings also moderated the effect of parental incarceration on the occurrence of risk behaviors.
Externalizing symptoms: Behavioral problems	Antle et al. (2019)^a^ [46]	(a) N = 3188 (b) M = 9 years ^b^ (c) PP = F	(d) TS = Long. (e) CV = 15–20	- Paternal incarceration was directly and significantly related to more significant behavior problems in boys and girls compared to the comparison group. - Mediation: Incarceration also indirectly affected behavioral problems through the mediation of maternal depression, the frequency of spankings, and parental involvement.
Externalizing symptoms: Aggressive behavior with peers	Dallaire and Zeman (2013) [49]	(a) N = 210 (b) M = 7–11 years (c) PP = B	(d) TS = Cross. (e) CV = 5–10	- Parental incarceration was significantly associated with higher levels of aggressiveness in children’s social relationships. - Moderation: Considering the children’s levels of empathy, the relationship was no longer significant for those with high empathy levels. Empathy acted as a moderator of the relationship.
Externalizing symptoms: Rule breaking behaviors	Del Toro et al. (2022) ^a^ [47]	(a) N = 4327 (b) M = 9 years ^b^ (c) PP = F	(d) TS = Long. (e) CV = 10	- Children who had experienced paternal incarceration between the ages of five and nine showed higher rule-breaking behaviors at nine. - Mediation: This relationship was partially mediated by children’s depressive symptoms.
Externalizing symptoms: Externalizing symptoms	Haskins (2015)^a^ [43]	(a) N = 2150 (b) M = 9 years ^b^ (c) PP = F	(d) TS = Long. (e) CV ≥ 20	- Paternal incarceration significantly predicted externalizing problems in children compared with those who had not been exposed to this phenomenon. - Moderation: When considering gender differences, a negative impact was observed for boys but not for girls.
Externalizing symptoms: Externalizing symptoms	Haskins and McCauley (2019)^a^ [41]	(a) N = 1664 (b) M = 9 years (c) PP = B	(d) TS = Long. (e) CV = 5–10	- Parental incarceration was significantly associated with greater child-reported and parent-reported externalizing problems.
Externalizing symptoms: Externalizing symptoms	Turney (2017)^a^ [17]	(a) N = 3065 (b) M = 9 years ^b^ (c) PP = F	(d) TS = Long. (e) CV ≥ 20	- A significant relationship was observed between parental incarceration and externalizing symptoms in children. - This result was also found within three groups with different probabilities of risk of entering prison (low, medium, and high).
Externalizing symptoms: Externalizing symptoms	Turney and Wildeman (2015)^a^ [18]	(a) N = 3197 (b) M = 9 years ^b^ (c) PP = M	(d) TS = Long. (e) CV ≥ 20	- This study did not find a significant relationship between maternal incarceration and externalizing problems in their children, compared to the comparison group. - Moderation (separation of groups without a proper test of moderation): When mothers were divided into three groups with a different probability of risk of entering prison (low, medium, and high), a significant relationship was observed between maternal incarceration and a greater presence of externalizing problem behaviors in the lowest risk stratum, but not in the medium and highest risk strata.
Externalizing symptoms: Externalizing symptoms	Wilbur et al. (2007) ^a^ [48]	(a) N = 102 (b) M = 9.5–11 years (c) PP = F	(d) TS = Long. (e) CV = 5–10	- Paternal incarceration significantly predicted externalizing problems in children.
Internalizing symptoms: Internalizing symptoms	25. Antle et al. (2019)^a^ [46]	(a) N = 3188 (b) M = 9 years ^b^ (c) PP = F	(d) TS = Long. (e) CV = 15–20	- The relationship between paternal incarceration and children presenting more internalizing symptoms was not directly significant. - Mediation: Paternal incarceration was found to exert a small but significant positive indirect effect on internalizing symptoms through parental characteristics (maternal depression and stress).
Internalizing symptoms: Childhood trauma	Arditti and Savla (2013) [50]	(a) N = 45 (b) M = 10.52–10.61 years ^b^ (c) PP = B	(d) TS = Cross. (e) CV = 10–15	- Parental incarceration was significantly associated with childhood trauma symptoms. - Mediation: In addition, the relationship between incarceration and parents’ perceptions of childhood trauma was mediated by problems during visits to the parents in prison.
Internalizing symptoms: Depressive symptoms	Del Toro et al. (2022)^a^ [47]	(a) N = 4327 (b) M = 9 years ^b^ (c) PP = F	(d) TS = Long. (e) CV = 10	- Children who experienced parental imprisonment between the ages of five and nine had more depressive symptoms at age nine than children who had not been exposed to parental imprisonment.
Internalizing symptoms: Internalizing symptoms	Haskins (2015)^a^ [43]	(a) N = 2150 (b) M = 9 years ^b^ (c) PP = F	(d) TS = Long. (e) CV ≥ 20	- Paternal incarceration significantly predicted internalizing problems in children. - Moderation: When considering differences in terms of gender, this effect was only maintained for boys.
Internalizing symptoms: Internalizing problems	Haskins and McCauley (2019)^a^ [41]	(a) N = 1664 (b) M = 9 years ^b^ (c) PP = B	(d) TS = Long. (e) CV = 5–10	- Parental incarceration was significantly associated with children reporting more internalizing problems.
Internalizing symptoms: Internalizing symptoms	Turney (2017)^a^ [17]	(a) N = 3065 (b) M = 9 years ^b^ (c) PP = F	(d) TS = Long. (e) CV ≥ 20	- A significant relationship was observed between parental incarceration and internalizing symptoms in children. - When fathers were divided into three groups with different probabilities of risk of entering prison (low, medium, and high), a significant relationship between paternal incarceration and a higher probability of presenting internalizing behaviors in the lowest and medium strata, but not in the highest stratum, was observed.
Internalizing symptoms:: Internalizing symptoms	Turney and Wildeman (2015)^a^ [18]	(a) N = 3197 (b) M = 9 years ^b^ (c) PP = M	(d) TS = Long. (e) CV ≥ 20	- Maternal incarceration was not a predictor of children’s internalizing symptoms. - Moderation (separation of groups without a proper test of moderation): When mothers were divided into three groups with a different probability of risk of entering prison (low, medium, and high), a significant relationship between maternal incarceration and a greater presence of externalizing problem behaviors in the lowest risk stratum, but not in the medium and highest risk strata was observed.
Internalizing symptoms: Depression	Wilbur et al. (2007) ^a^ [48]	(a) N = 102 (b) M = 9.5–11 years (c) PP = F	(d) TS = Long. (e) CV = 5–10	- A significant relationship was found between paternal incarceration and children’s depressive symptoms (when children reported these symptoms, but not when parents did).
Material hardship: Economic risks	Geller et al. (2011) [51]	(a) N = 3469 (b) M = 9 years ^b^ (c) PP = F	(d) TS = Long. (e) CV = 15–20	- A statistically significant relationship was observed between paternal incarceration and higher economic risks for their children. - Mediation: The indirect mechanisms through which this phenomenon occurred were the relationship between parents, the labor market performance, and the fact that the father did not reside with the family prior to incarceration.
Material hardship: Father’s financial contributions	Washington et al. (2018) [52]	(a) N = 1185 (b) M = 9 years ^b^ (c) PP = F	(d) TS = Long. (e) CV ≥ 20	- A significant relationship was observed between paternal incarceration and a lower economic contribution to child support.

N = Size; M = Mean Age; PP = Parent in Prison; TS = Type of Study; CV = Number of Control Variables; F = Father; M = Mother; B = Both^;^ Cross. = Cross Sectional; Long. = Longitudinal^; a^ Studies that include different types of criterion variables or focus on more than one developmental stage.; ^b^ Mean age is not shown, only the age of the children at the time of the assessment.

**Table A3:** Summary of studies included in the systematic review. Adolescents aged 12 to 18 years old.

Criterion Variable	Study	Characteristics of the Study:		Results
		(a) Size (b) Mean Age (c) Parent in Prison	(d) Type of Study (e) Control Variables	
Cognitive skills and academic performance: Attentional difficulties	Boch et al. (2019) [15]	(a) N = 613 (b) M = 14.5 years ^c^ (c) PP = B	(d) TS = Cross. (e) CV = 5–10	The relationship between paternal incarceration and attention difficulties in adolescents was not significant when considering possible adverse childhood experiences.
Cognitive skills and academic performance: Educational attainment and school absenteeism	Brown (2016) [55]	(a) N = 103,536 (b) M = 15.81 years ^c^ (c) PP = M	(d) TS = Cross. (e) CV = 15–20	An association was found between maternal incarceration and academic outcomes. The moment of incarceration according to the child’s age and the difference between compulsory and non-compulsory education (specifically, colleg(e) were important determinants of these variable effects. Maternal incarceration predicted compulsory educational achievement during adolescence more prominently when it occurred between birth and age four (increased grade repetition) and between ages five and ten (increased high school dropouts). In both cases, the effects were persistent and occurred years after incarceration.
Cognitive skills and academic performance: Academic performance, involvement in fights, school absenteeism, and membership	McCauley (2020) [53]	(a) N = 11,767 M = 15.77 years ^d^ (c) PP = B	(d) TS = Long. (e) CV = 5–10	Participants who had suffered parental incarceration experienced lower rates of achieving B grades or better. There was a relationship between paternal (or maternal) incarceration and lower academic performance in English (not in mathematics). Paternal incarceration was significantly associated with higher odds of failing grades, being expelled, being involved in fights, school absenteeism, not participating in school activities, and not feeling part of the school. In addition, maternal incarceration was associated with a greater likelihood of getting into fights and school absenteeism.
Cognitive skills and academic performance: Academic performance	Murray et al. (2012) ^a^ [47]	(a) N = 1009 (b) M = 7–19 years (c) PP = B	(d) TS = Long. (e) CV = 10–15	No significant relationship was found between parental incarceration and academic performance.
Cognitive skills and academic performance: Truancy, the highest level of education, and cumulative academic achievement	Nichols et al. (2016) [58]	N = 71,447 (truancy); 69,082 (highest level of education); 46,045 (cumulative academic achievement) (b) M = 15.9 years (c) PP = B	(d) TS = Cross. (e) CV = 10–15	Parental incarceration was significantly associated with school absenteeism, academic performance, and higher educational attainment, with the strongest association being school absenteeism. Moderation (parental incarceration as moderator): The relationship between higher school connectedness and higher educational achievement in children disappeared in cases where the children had a parent in prison.
Cognitive skills and academic performance: Failure to complete secondary school and prolonged school absenteeism	Nichols and Loper (2012) [57]	(a) N = 3338 (b) M = 26.5 years, study date (asked about adolescence) (c) PP = HM	(d) TS = Long. (e) CV ≤ 5	Adolescents who experienced the incarceration of an extended family member living in their household before the age of eighteen were more likely to report a prolonged absence from school (more than 30 days) and not to finish high school than those who did not report having a household member in prison. This relationship was not found when a parent was the one in prison, although a statistical trend was observed.
Cognitive skills and academic performance: Achievement, discipline, school connectedness, and engagement	Shlafer et al. (2017) [59]	(a) N = 114,828 (b) M = 14.90 years ^c^ (c) PP = B	(d) TS = Cross. (e) CV = 5–10	Parental incarceration was significantly associated with students’ poor school-based outcomes. Moderation: Parental incarceration was associated with different school outcomes by school setting. Among youth in public school settings, parental incarceration was consistently associated with poor school outcomes (lower levels of achievement, less engagement and connectedness, and a greater likelihood of receiving disciplinary action than peers who never experienced parental incarceration).
Cognitive skills and academic performance: Attention problems	Turney (2022) ^a^ [54]	(a) N = 3416 (b) M = 15.59 years (c) PP = F	(d) TS = Long. (e) CV ≥ 20	Paternal incarceration was associated with more attentional difficulties (adjusted estimates). Moderation (separation of groups without a proper moderation test): Paternal incarceration in early childhood was associated with more attention problems but not in middle childhood and adolescence (adjusted estimates). Mediation: The relationship between early childhood paternal incarceration and attention problems was significantly mediated by parental relationship, economic well-being, parenting style, health, and type of residence (introduced all mechanisms together in the analysis).
Socioemotional skills: Prosocial behavior	Bradshaw et al. (2021)^a^ [61]	(a) N = 8568 (b) M = 13 years ^b^ (c) PP = B	(d) TS = Long. (e) CV = 5	No direct relationship was observed between parental incarceration and prosocial behavior at age 13. Mediation: The relationship between having a parent in prison at age nine and presenting worse prosocial behavior at age thirteen was mediated by higher levels of caregiver depression and poor caregiver–child relationship quality.
Socioemotional skills: Social network size and location	Bryan (2017) [60]	(a) N = 11,356 (b) M = 14.9 years (c) PP = F	(d) TS = Long. (e) CV = 10–15	Adolescents with a father in prison showed more peripheral social relationships than their peers. Specifically, they showed lower centrality positions, smaller extensive networks, and their friends were less advantaged and academically successful in school and committed more delinquent acts.
Socioemotional skills: Social networks size and location, participation in antisocial contexts	Cochran et al. (2018) [62]	(a) N = 11,681 (b) M = 14.99 years (c) PP = B	(d) TS = Long. (e) CV = 5–10	Inconsistent results were found on the relationship between parental incarceration and adolescents’ social relationships. Overall, no significant association was found, but specifically, parental incarceration negatively affected the youth’s social network characteristics (friends with lower grades who lied more, missed more school, and got into fights). No relationship was found between parental incarceration and school integration.
Risk behaviors: Substance abuse	Bomysoad and Francis (2021)^a^ [14]	(a) N = 29,617 (b) M = 12–17 years (c) PP = B	(d) TS = Cross. (e) CV = 5–10	Parental incarceration significantly predicted greater substance abuse problems in adolescents, Moderation: Resilience and sleep quality moderated this relationship.
Risk behaviors: Alcohol, tobacco, marihuana use, other drugs	Davis and Shlafer (2017)^a^ [65]	(a) N = 122,180 (b) M = 14.87 years (c) PP = B	(d) TS = Cross. (e) CV = 5–10	Parents’ past and present incarceration were significantly associated with alcohol, tobacco, and other substance use and abuse in adolescents. Across all outcomes, adolescents with incarcerated or released parents had higher rates of substance abuse than children without an incarcerated parent. In addition, youth with currently incarcerated parents performed worse than their peers with a history of parental incarceration on nearly every indicator.
Risk behaviors: AIDS/HIV-related drug use and sexual risk	Khan et al. (2018) [66]	(a) N = 11,884 (b) M = 15.9 years (c) PP = B	(d) TS = Long. (e) CV = 5–10	Parental incarceration was associated with higher marijuana use by adolescents, especially in those who had experienced parental incarceration before the age of eight. On the other hand, parental incarceration only significantly predicted STD acquisition for black children.
Risk behaviors: Substance use (alcohol and tobacco)	Kinner et al. (2007) ^a^ [63]	(a) N = 2339 (b) M = 14 years (c) PP = F	(d) TS = Long. (e) CV = 5–10	No association was observed between parental incarceration and externalizing problems as well as substance use in adolescence. For boys, paternal detention but not incarceration predicted alcohol abuse.
Risk behaviors: Marijuana use	Murray et al. (2012) ^a^ [47]	(a) N = 1009 (b) M = 7–19 years (c) PP = B	(d) TS = Long. (e) CV = 10–15	There was no significant relationship between parental incarceration and marijuana use.
Risk behaviors: Early sexual onset	Turney and Goldberg (2019) [64]	(a) N = 3405 (b) M = 15.6 years (c) PP = F	(d) TS = Long. (e) CV ≥ 20	Paternal incarceration was positively associated with early sexual onset. Moderation: Consequences of paternal incarceration on early sexual onset were stronger among boys who lived with their fathers prior to incarceration compared to girls. Mediation: This relationship occurred directly and indirectly through externalizing problems as a mediator.
Delinquent behaviors: Serious youth delinquency	Kjellstrand and Eddy (2011) [68]	(a) N = 655 (b) No M: 5th, 8th, and 10th grade (c) PP = B	(d) TS = Long. (e) CV = 5–10	No direct relationship was found between paternal incarceration and juvenile delinquency Mediation: This relationship did occur indirectly, mediated by other variables related to social disadvantages, the mental health of the parents, and the effectiveness of parenting.
Delinquent behaviors: Theft	Murray et al. (2012) ^a^ [47]	(a) N = 1009 (b) M = 7–19 years (c) PP = B	(d) TS = Long. (e) CV = 10–15	An association was found between parental incarceration and a temporary increase in theft. This increase occurred when the children lived with their parents before the incarceration.
Delinquent behaviors: Material or monetary gain	Porter and King (2015) [67]	(a) N = 2283 (b) M = 15.56 years (c) PP = F	(d) TS = Long. (e) CV = 10–15	No direct relationship was found between paternal incarceration and juvenile delinquency. Mediation: Paternal incarceration was significantly related to expressive delinquency (crimes resulting from anger or frustration) through the reduction of attachment to fathers.
Delinquent behaviors: Delinquent behaviors	Turney (2022)^a^ [54]	(a) N = 3416 (b) M = 15.59 years (c) PP = F	(d) TS = Long. (e) CV ≥ 20	Paternal incarceration was significantly associated with juvenile delinquency. Moderation (separation of groups without a proper test of moderation): Paternal incarceration in early childhood was associated with more juvenile delinquency but not in middle childhood or adolescence (adjusted estimates).
Externalizing symptoms: Externalizing symptoms	Boch et al. (2019) ^a^ [15]	(a) N = 613 (b) M = 14.55 years ^c^ (c) PP = F	(d) TS = Cross. (e) CV = 5–10	Parental incarceration did not significantly predict externalizing problems in adolescents when other adverse childhood experiences were included.
Externalizing symptoms: Behavioral problems	Bomysoad and Francis (2021)^a^ [14]	(a) N = 29,617 (b) M = 12–17 years (c) PP = B	(d) TS = Cross. (e) CV = 5–10	Parental incarceration significantly predicted behavioral problems in adolescents. This relationship was moderated by the adolescents’ resilience and engagement in extracurricular activities.
Externalizing symptoms: Behavioral problems	Bradshaw et al. (2021)^a^ [61]	(a) N = 8568 (b) M = 13 years ^b^ (c) PP = B	(d) TS = Long. (e) CV = 5	No significant direct relationship was found between parental incarceration at age nine and behavioral problems at age thirteen. Mediation: Having a father in prison at age nine predicted higher levels of depression in the caregiver, which in turn implied a worse relationship between the caregiver and the child and ultimately led to greater behavioral problems.
Externalizing symptoms: Rule-breaking behavior	Del Toro et al. (2022) ^a^ [47]	(a) N = 4327 (b) M = 15 years ^b^ (c) PP = F	(d) TS = Long. (e) CV = 10	Children who experienced parental incarceration at age five or nine had higher norm noncompliance at age fifteen than children who had not been exposed to parental incarceration. Mediation: This relationship was partially mediated by children’s depressive symptoms.
Externalizing symptoms: Behavioral problems	Davis and Shlafer (2016) ^a^ [12]	(a) N = 122,180 (b) M = 14.87 years (c) PP = B	(d) TS = Cross. (e) CV = 5–10	Having an incarcerated parent was significantly associated with greater behavioral problems in adolescents, twice as high as peers without an incarcerated parent. Moderation: The protective effect of parental closeness was strongest for children with no experience of parental incarceration compared to children with former and current experience. However, its protective effect was significant in the three groups.
Externalizing symptoms: Behavioral problems	47. Kinner et al. (2007) ^a^ [63]	(a) N = 2399 (b) M = 14 years ^b^ (c) PP = F	(d) TS = Long. (e) CV = 5–10	No association was found between parental incarceration and externalizing problems.
Externalizing symptoms: Externalizing symptoms	Kjellstrand; et al. (2018) [69]	(a) N = 361 (b) M = 10–16 years (c) PP = B	(d) TS = Long. (e) CV = 5–10	Parental incarceration was significantly related to externalizing symptoms in adolescence.
Externalizing symptoms: Externalizing symptoms	Kjellstrand et al. (2019) [9]	(a) N = 655 (b) No M: 10, 12, 14, and 16 years (c) PP = B	(d) TS = Long. (e) CV = 5–10	Parental incarceration was a significant predictor of externalizing symptoms in one of the problematic trajectory groups named “Mid-Increasing trajectory”. This group is described by the following characteristics: low externalizing problems at age ten, but gradually increasing to clinically high levels at age sixteen.
Externalizing symptoms: Behavioral problems	Philips et al. (2002) ^a^ [70]	(a) N = 258 (b) M = 13.7 years (c) PP = B	(d) TS = Long. (e) CV = 10–15	Adolescents who experienced a parent’s incarceration showed significantly more behavioral problems.
Externalizing symptoms: Externalizing symptoms	Turney (2022) ^a^ [54]	(a) N = 3416 (b) M = 15.59 years (c) PP = F	(d) TS = Long. (e) CV ≥ 20	Paternal incarceration was associated with more externalizing problems (adjusted estimates). Moderation (separation of groups without a proper moderation test): Paternal incarceration in early childhood was associated with more externalizing problems, but not in middle childhood and adolescence (adjusted estimates). Mediation: The relationship between early childhood paternal incarceration and externalizing problems was significantly mediated by parental relationship, economic well-being, parenting, health, and residence (introduced all mechanisms together in the analysis).
Internalizing symptoms: Internalizing symptoms	Boch et al. (2019) [15]	(a) N = 613 (b) M = 14.55 years ^c^ (c) PP = F	(d) TS = Cross. (e) CV = 5–10	No significant relationship was found between parental incarceration and internalizing symptoms.
Internalizing symptoms: Emotional distress	Bradshaw et al. (2021)^a^ [61]	(a) N = 8568 (b) M = 13 years ^b^ (c) PP = B	(d) TS = Long. (e) CV = 5	No significant direct relationship was found between parental incarceration at age nine and experiencing emotional distress at age thirteen. Mediation: There was an indirect relationship between parental incarceration and emotional distress through the caregiver’s depression.
Internalizing symptoms: Depression, anxiety, ADHD	Bomysoad and Francis (2021) ^a^ [14]	(a) N = 29,617 (b) M = 12–17 years (c) PP = B	(d) TS = Cross. (e) CV = 5–10	Parental incarceration significantly predicted higher mental health problems in adolescents (anxiety, depression, ADHD, behavioral problems, and substance abus(e) than in adolescents without an incarcerated parent. Moderation: Different moderating variables were found for the relationship between parental incarceration and internalizing symptoms, such as resilience, extracurricular activity, physical activity, sleep quality, or screen time.
Internalizing symptoms: Mental health and emotional problems	Davis and Shlafer (2016) ^a^ [12]	(a) N = 122,180 (b) M = 14.87 years (c) PP = B	(d) TS = Cross. (e) CV = 5–10	Children of incarcerated parents showed poorer mental health than the rest of the adolescents, with those of currently incarcerated parents at the highest risk. Adolescents who had been exposed to previous parental incarceration were approximately twice as likely to experience them, while those whose parent was currently in prison were between two and a half and four times as likely. Moderation: Parental closeness acted as a protective factor of the relationship between parental incarceration and mental health. This protective effect was strongest for children with no experience of parental incarceration compared to children with former and current experiences, although it was significant in the three groups.
Internalizing symptoms: Depressive symptoms	Del Toro et al. (2022) ^a^ [47]	(a) N = 4327 (b) M = 15 years ^b^ (c) PP = F	(d) TS = Long. (e) CV = 10	Children who experienced parental imprisonment at age five or nine had more depressive symptoms at age fifteen than children who had not been exposed to parental imprisonment.
Internalizing symptoms: Internalizing symptoms	Kinner et al. (2007) ^a^ [63]	(a) N = 2339 (b) M = 14 years ^b^ (c) PP = F	(d) TS = Long. (e) CV = 5–10	The relationship between parental incarceration and internalizing symptoms in children was not significant.
Internalizing symptoms: Internalizing symptoms	Kjellstrand et al. (2020) [71]	(a) N = 671 (b) M = 10–16 years (c) PP = B	(d) TS = Long. (e) CV = 5–10	No significant relationship was found between parental incarceration and internalizing problems.
Internalizing symptoms: Depression	Murray et al. (2012)^a^ [56]	(a) N = 1009 (b) M = 7–19 years (c) PP = B	(d) TS = Long. (e) CV = 10–15	No association was found between parental incarceration and depression in adolescents.
Internalizing symptoms: Mental health	Philips et al. (2002) ^a^ [70]	(a) N = 258 (b) M = 13.7 years (c) PP = B	(d) TS = Long. (e) CV = 10–15	Adolescents who experienced parental incarceration showed significantly more ADHD and lower levels of depression.
Internalizing symptoms: Post-traumatic stress and general psychological problems	Shehadeh et al. (2015) [72]	(a) N = 314 (b) M = 13.4 years (c) PP = F	(d) TS = Cross. (e) CV = 5–10	Paternal incarceration was significantly associated with greater mental health problems in their children, compared to the comparison group. Additionally, witnessing the father’s arrest increased these mental health problems in this group of children.
Internalizing symptoms: Internalizing symptoms	Turney (2022)^a^ [54]	(a) N = 3416 (b) M = 15.59 years (c) PP = F	(d) TS = Long. (e) CV ≥ 20	Paternal incarceration was not associated with more internalizing problems (adjusted estimates).

N = Size; M = Mean Age; PP = Parent in Prison; TS = Type of Study; CV = Number of Control Variables; F = Father; M = Mother; B = Both; HM = Household Member (Parent, Sibling, Extended Family)^;^ Cross. = Cross Sectional; Long. = Longitudinal^; a^ Studies that include different types of criterion variables or focus on more than one developmental stage. ^b^ Mean age is not shown, only the age of the children at the time of the assessment. ^c^ Mean age was calculated from the mean age of each subgroup, considering their sample size. ^d^ Mean age was calculated from the mean age of each subgroup without considering their sample size because it was not reported.

**Table A4:** Summary of studies included in the systematic review. Studies that do not differentiate according to children’s and adolescents’ developmental stage.

Criterion Variable	Study	Characteristics of the Study		Results
		(a) Size (b) Mean Age (c) Parent in Prison	(d) Type of Study (e) Control Variables	
Physical health: General physical health and chronic physical conditions	Jackson et al. (2021) ^a^ [5]	(a) N = 102,341 (b) M = 0–17 years (c) PP = B	(d) TS = Cross. (e) CV = 10	- Parental incarceration was significantly associated with worse physical health and chronic disease problems.
Physical health: Oral Health	Testa and Jackson (2020) [73]	(a) N = 99,962 (b) M = 9.65 years ^c^ (c) PP = B	(d) TS = Cross. (e) CV = 5–10	- Children whose parents had ever been in prison had lower oral health, including weak or fair teeth, toothaches, gum bleeding, cavities, and tooth decay, and they were also more likely to have unmet dental care needs. - Moderation: Attenuation analyses indicated that this relationship partially accounted for household material hardship and children’s health insurance.
Physical Health: General and physical health, activity, and school absenteeism	Turney (2014) ^a^ [4]	(a) N = 95,677 (b) M = 0–17 years (c) PP = B	(d) TS = Cross. (e) CV = 20	- No significant relationship was found between parental incarceration and physical health measures.
Physical Health: Unmet medical needs	Turney (2017) ^a^ [41]	(a) N = 95,531 (b) M = 0–17 years (c) PP = B	(d) TS = Cross. (e) CV ≥ 20	- Children exposed to parental incarceration were 26% more likely to have unmet healthcare needs. - When the different medical needs were studied separately, the difference was not significant for physical health measures.
Physical Health: Mortality	Wildeman et al. (2014) [74]	(a) N = 58,848 (b) M = 0–20 years (c) PP = B	(d) TS = Long. (e) CV = 15–20	- Moderation (separation of groups without a proper moderation test): Paternal and maternal imprisonment were associated with higher male child mortality, whereas paternal imprisonment was associated with lower child mortality risks for girls. There was no clear association between maternal incarceration and female child mortality.
Internalizing symptoms: Mental health and developmental problems	Jackson et al. (2021) ^a^ [5]	(a) N = 102,341 (b) M = 0–17 years (c) PP = B	(d) TS = Cross. (e) CV = 10	- Parental incarceration was significantly associated with children’s general mental health problems.
Internalizing symptoms: Mental health	Turney (2014) ^a^ [4]	(a) N = 95,677 (b) M = 0–17 years (c) PP = B	(d) TS = Cross. (e) CV = 20	- Parental incarceration was significantly associated with five child health variables: learning disabilities, ADD/ADHD, behavioral problems, developmental delays, and language disorders.
Internalizing symptoms: Mental health	Turney (2017) ^a^ [75]	(a) N = 95,531 (b) M = 0–17 years (c) PP = B	(d) TS = Cross. (e) CV ≥ 20	- Children exposed to parental incarceration were 26% more likely to have unmet healthcare needs. Children with an incarcerated parent were 60% more likely to have a mental health problem than other children.

N = Size; M = Mean Age; PP = Parent in Prison; TS = Type of Study; CV = Number of Control Variables; F = Father; M = Mother; B = Both; Cross. = Cross Sectional; Long. = Longitudinal; ^a^ Studies that include different types of criterion variables or focus on more than one developmental stage. ^c^ Mean age was calculated from the mean age of each subgroup, considering their sample size.
